# Sleep Position Detection with a Wireless Audio-Motion Sensor—A Validation Study

**DOI:** 10.3390/diagnostics12051195

**Published:** 2022-05-11

**Authors:** Wojciech Kukwa, Tomasz Lis, Jonasz Łaba, Ron B. Mitchell, Marcel Młyńczak

**Affiliations:** 1 Faculty of Dental Medicine, Medical University of Warsaw, 02-091 Warsaw, Poland; 2 Department of Pediatric ENT, Medical University of Warsaw, 02-091 Warsaw, Poland; lis.tomasz.lis@gmail.com; 3 Institute of Metrology and Biomedical Engineering, Faculty of Mechatronics, Warsaw University of Technology, 02-525 Warsaw, Poland; jonasz.laba.dokt@pw.edu.pl (J.Ł.); marcel.mlynczak@pw.edu.pl (M.M.); 4 Department of Otolaryngology, UT Southwestern Medical Center, Dallas, TX 75390, USA; ron.mitchell@utsouthwestern.edu

**Keywords:** home sleep study, polysomnography, actigraphy, positional sleep apnea

## Abstract

It is well documented that body position significantly affects breathing indices during sleep in patients with obstructive sleep apnea. They usually worsen while changing from a non-supine to a supine position. Therefore, body position should be an accurately measured and credible parameter in all types of sleep studies. The aim of this study was to specify the accuracy of a neck-based monitoring device (Clebre, Olsztyn, Poland) mounted at the suprasternal notch, in determining a supine and non-supine sleeping position, as well as specific body positions during sleep, in comparison to polysomnography (PSG). A sleep study (PSG along with a neck-based audio-motion sensor) was performed on 89 consecutive patients. The accuracy in determining supine and non-supine positions was 96.9%±3.9% and 97.0%±3.6%, respectively. For lateral positions, the accuracy was 98.6%±2% and 97.4%±4.5% for the right and left side, respectively. The prone position was detected with an accuracy of 97.3%±5.6%. The study showed a high accuracy in detecting supine, as well as other gross positions, during sleep based on a sensor attached to the suprasternal notch, compared to the PSG study. We feel that the suprasternal notch is a promising area for placing wireless sleep study devices.

## 1. Introduction

Obstructive sleep apnea (OSA) is the most common form of sleep-disordered breathing (SDB), characterized by repeated episodes of absent (apnea) or reduced (hypopnea) airflow in the upper airway during sleep. Airway obstruction is associated with either oxygen desaturation or frequent brain arousal and is linked to increased incidence of hypertension, type 2 diabetes, atrial fibrillation, heart failure, coronary artery disease, stroke, and death [1,2].

The gold standard test for diagnosis of OSA is a laboratory-based attended polysomnography (PSG) during which multiple data channels are recorded, including sleep and respiratory parameters, muscle activity, heart rhythm, snoring, and body position. The presence and severity of OSA are typically determined by the apnea–hypopnea index (AHI), defined as the number of apneas and hypopneas per hour of sleep. It is generally accepted that AHI ≥ 5 events/h defines OSA.

It is well documented that body position significantly affects breathing indices, expressed among others by the AHI, which usually worsens while changing from a non-supine to a supine position [3]. Therefore, body position should be an accurately measured and credible parameter. Positional obstructive sleep apnea (POSA), first described by Cartwright in 1984 [3], is defined as an AHI at least twice as high in the supine position as in the lateral (non-supine) position [4]. According to Cartwright’s criteria, two subtypes of POSA were defined: supine-isolated OSA (siOSA; non-supine AHI < 5 with at least 15 min of sleep in both positions) and supine-predominant OSA (spOSA; non-supine AHI ≥ 5) [5,6,7]. In selected patients with POSA, positional therapy (PT), which is based on preventing patients from sleeping in a supine position, may be an effective treatment option [8,9]. Ravesloot et. al. provided an overview of 16 articles, examining the impact of PT on OSA, showing that all included studies reported a positive effect of PT on AHI reduction in POSA patients [9].

As mentioned before, for the clinical needs of sleep study analysis, body position can be divided into supine and non-supine, which consists of prone and both lateral positions. Body position sensors are usually attached to either the chest or abdomen, which does not always reflect the actual orientation of the upper airways. Furthermore, current American Academy of Sleep Medicine (AASM) guidelines, except for frequency of measurement, do not provide recommendations concerning the recording of body position during sleep. To our knowledge, there is also a lack of comparison studies between different PSG systems, moreso regarding the accuracy of detecting body position.

Furthermore, full PSG is a very uncomfortable, expensive, and availability-limited study. Lately, multiple sensors have been introduced into sleep medicine studies with a promise to diagnose sleep disordered breathing in the home environment. These cheaper and more comfortable than in-lab PSG devices belong mostly to home sleep apnea tests (HSATs). Here we used a small audio-motion wireless sensor (Clebre, Olsztyn, Poland). Clebre is attached to the skin at the suprasternal notch on the neck with a double-sided medical patch (see Section 2), allowing detection of the patient’s position and activity. Previously we showed a very high accuracy in detecting a supine and non-supine sleep position in comparison to PSG on a group of 30 patients [10].

The aim of this study was to validate the accuracy of a neck-based Clebre device in determining a supine and non-supine sleeping position, as well as specific body positions during sleep, in comparison to the NOX A1 PSG system (Nox Medical Inc., Reykjavik, Iceland) in a large cohort of patients.

## 2. Materials and Methods

### 2.1. Participants

The study included 89 consecutive adult patients who underwent PSG and Clebre examinations. The inclusion criteria were: 18 years of age or older, PSG for suspected OSA, at least 6 h of simultaneously recorded PSG and Clebre. The exclusion criteria were a previous history of OSA treatment such as positive airway pressure (PAP) therapy and class III and IV heart failure according to the classification of the New York Heart Association (NYHA) [11].

All participants signed an informed consent. The study was approved by the Ethics Committee of Medical University of Warsaw (KB/14/2018).

Demographic information, including age, sex, height, and weight, was collected. The body mass index (BMI) was calculated for each patient.

### 2.2. Protocol and Devices

Each subject underwent a full-night attended PSG in the sleep laboratory of the Otorhinolaryngology Department at Czerniakowski Hospital, Warsaw, Poland. PSGs were recorded with the Nox A1 PSG System [12,13]. The recording montage comprised a 6-channel encephalogram (EEG), a 3-channel submental electromyogram (EMG), a left and right electrooculogram (EOG), an electrocardiogram (ECG), airflow recording through the nose and mouth by a nasal air pressure transducer and oronasal thermistor, thoracic and abdominal effort measurement by inductance plethysmography, and arterial oxygen saturation using a Nonin 3150 WristOx2™ wireless oximeter (Nonin Medical, Plymouth, MN, USA) [14]. An in-built microphone was used to record snoring. Body positions, differentiated between upright, right side, left side, prone, and supine, were determined by a 3-axis, ±2 g accelerometer with 20 Hz sampling frequency, incorporated in the PSG headbox. The video was recorded throughout the night using AXIS M3106-LVE [15] and AXIS M3116-LVE [16] network cameras (Axis Communications AB, Lund, Sweden) with 1024×768 resolution and a frame rate of 30.

For each patient, sleep and respiratory events were scored by an experienced sleep physician using the AASM Manual for the Scoring of Sleep and Associated Events v.2.6 [17]. The sleep position was obtained from the PSG’s raw data acquired from European Data Format (EDF) files, exported from the Noxturnal software (Nox Medical Inc., Reykjavik, Iceland). Sleep parameters calculated by the software were obtained from PSG sleep reports.

**Figure 1:**
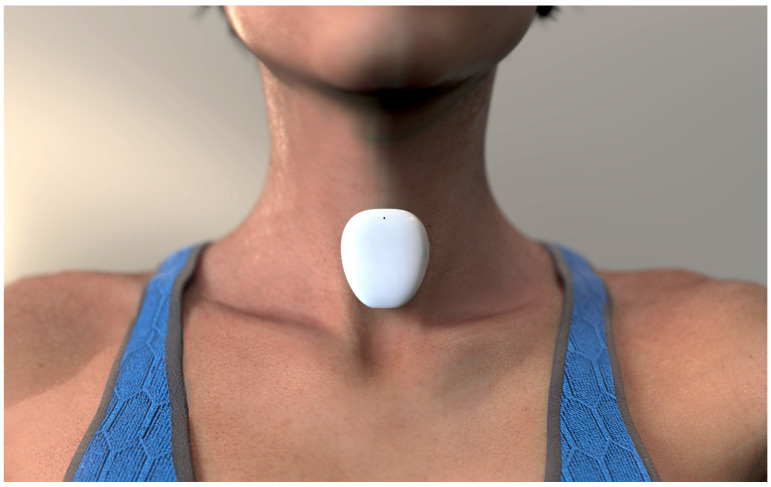
The placement of the Clebre audio and motion sensor.

Simultaneously, subjects underwent full night examination with a Clebre audio and motion sensor. The sensor was placed by the technician in the suprasternal notch on the neck and attached using a medical double-sided patch. The way the sensor was placed is presented in Figure 1. The dimensions of the sensor were 33×39×13 mm, and it weighed 18 g. The battery allowed for at least 14 h of operation. The memory capacity was defined internally by a 2 GB FLASH chip. Motion accelerometry based signals (3-axis, with a 52 Hz sampling frequency) were used to estimate sleep body position characteristics, using the algorithms presented in a previous study, which reported 97% accuracy in supine versus non-supine body position differentiation compared to the simultaneously acquired PSG [18]. The sleep studies in which there were differences in estimation of body position between PSG and Clebre were visually inspected by two PSG technicians, using video monitoring. To synchronize the devices, both the Clebre and PSG had an internal clock. It ran parallel to the connected computer (PSG) or was synchronized at the beginning of the study using a dedicated smartphone app (Clebre).

### 2.3. Data Analysis and Statistics

For each patient the percentage of supine/non-supine body position was recorded. Among POSA patients, subjects who spent less than 15 min of sleep in either supine or non-supine sleeping positions were excluded [7]. We analyzed differences between PSG and Clebre using paired t tests. Furthermore, Lin’s Concordance Coefficients [19] were estimated for each body position, and Bland–Altman analyses [20] were performed. All calculations were performed using Python 3.9.7 (default, 16 September 2021, 13:09:58), graphs were created using matplotlib package [21]. The significance level was established at the level of 0.05.

## 3. Results

Demographics of the study population are presented in Table 1 and OSA data in Table 2. There were 89 participants in the study, including 21 females. Their average age was around 50 and average BMI around 30. Almost all (95.5%) were considered OSA patients, from which 52.8% were POSA ones.

The accuracy in determining supine and non-supine positions was 96.9%±3.9% and 97.0%±3.6%, respectively. For lateral positions, the accuracy was 98.6%±2.0% and 97.4%±4.5% for the right and left side, respectively. Paired t tests suggested no basis to reject the null hypothesis (equal means) for left and right side body positions. The prone position was detected with an accuracy of 97.3%±5.6% (*p*-value = 0.0009). The *p*-value for supine (0.016) showed a statistically significant difference; however, their absolute values were relatively small.

**Figure 2:**
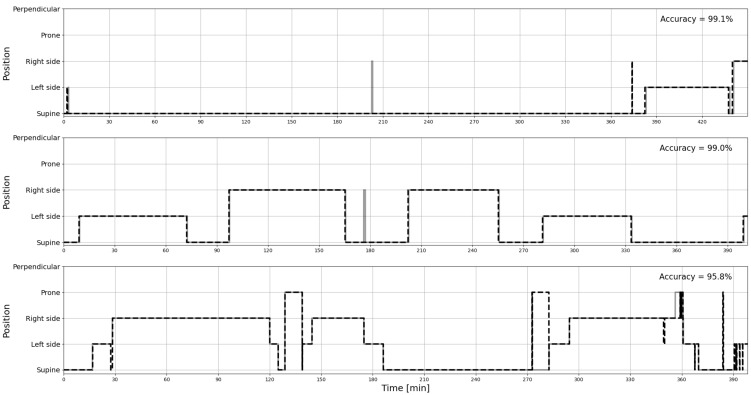
The sample curves of actigraphy signals recorded by PSG (solid line) and estimated from Clebre (dashed line), selected to show an almost perfect match (**top**), a small level of error (**middle**), and a moderate level of error (**bottom**), respectively.

The sample actigraphy curves for three patients were presented comparatively in Figure 2. Those were selected to show examples with almost identical signals, with a small level of error, and with a moderate level of error, respectively.

**Figure 3:**
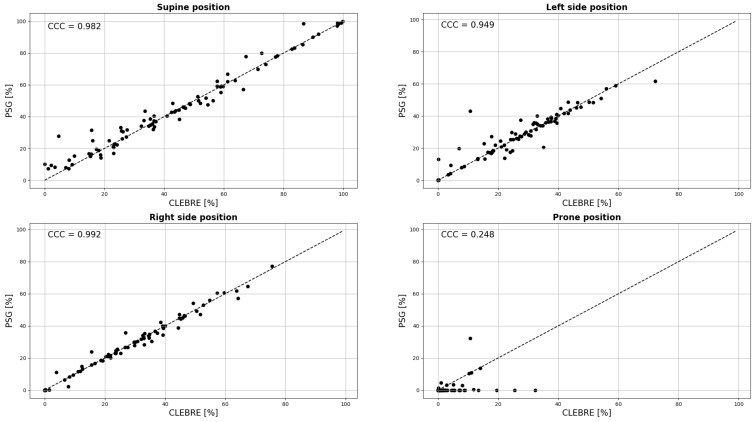
The PSG versus Clebre plots for percentages of each lying body position in relation to sleep time, along with the estimation of Lin’s Concordance Coefficients (CCC).

**Figure 4:**
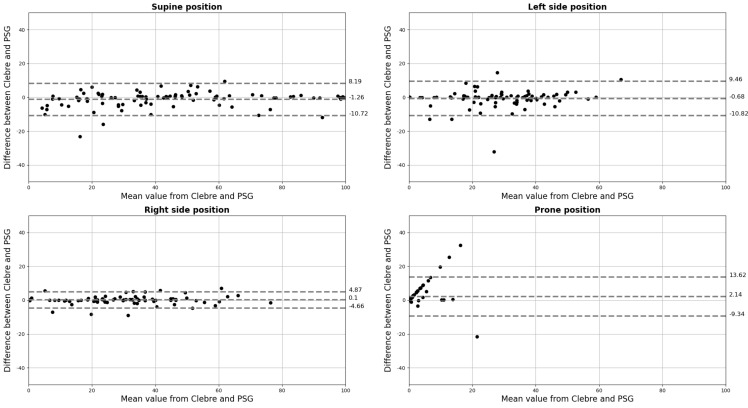
The Bland–Altman plots for percentages of each lying body position in relation to sleep time; percentages on both axes.

The Lin’s Concordance Coefficients and Bland–Altman curves were also prepared to compare PSG and Clebre percentages of each lying body position in relation to sleep time and are presented in Figure 3 and Figure 4, respectively.

## 4. Discussion

To our knowledge, this is the first study validating sleep position detection of any wireless HSAT sensor in a large cohort of patients against in-lab PSG, as such reports are scarce and all prepared on very limited number of patients [22,23,24].

According to a paper recently published by Ravesloot and colleagues, a standardized framework emphasizing the role of the sleeping position should be an important dataset in every sleep report [18,25]. This is because sleep position may significantly influence sleep study results, as well as the fact that a large group of OSA patients can benefit largely from PT. Therefore, accurate sleep position detection should be a crucial part of every sleep study.

Our study showed high accuracy in detecting both supine vs. non-supine and specific sleep positions with a wireless sensor attached to the skin at the suprasternal notch, in comparison to PSG, in a large cohort of patients. In terms of sleep study results, the binary division into supine and non-supine is of most importance. Here, the accuracy equaled 96.9%±3.9% and 97.0%±3.6%, respectively. In some patients where differences were noticed, they resulted mostly from discrepancies between head versus thorax orientation.

Every PSG system (type 1 and type 2 devices; the full classification is provided in [26,27]) measures and analyzes sleep position. According to AASM, the required sampling rate for body position measurement is 1 Hz [28]. Aside from that, there is no information regarding accuracy of measurement or angle-limit values in each axis for classification of body positions while asleep (supine, prone, and lateral positions). Interestingly, a study by Ferrer-Lluis et al. showed on 19 OSA patients that automatic sleep position determination in PSG agreed, on average, only in 83.1% with video-validated positions [23].

Unlike PSG, home sleep apnea testing (HSAT) which typically uses type 3 devices, are not required to record the sleep position [29,30], only “conventional” type 3 devices do measure these parameters [31]—for sensors without the measurement of sleep position it is impossible to confirm POSA and to implement PT. As there are on average 56% of patients with POSA [32,33,34,35] when using the most commonly used Cartwright’s definition, this parameter is absolutely crucial to include in the sleep study. Type 4 devices measure one or two variables: oxygen saturation, airflow, and chest movement, and in most cases they do not take body position into account, therefore their use in determining POSA is very limited.

There are many different new technologies that are being introduced in sleep medicine to assess body position. The devices in which these are introduced belong largely to HSAT. Among them, there are both contact and non-contact technologies. Non-contact technologies are bed mattresses and radar sensors. In terms of bed mattresses, the Sonomat is the most validated in sleep medicine [36,37]. No data regarding sleep position recognition are available in the published papers. For other mattress systems, there are studies showing a potentially high accuracy in detecting all four major sleep positions. The accuracy of a system described by Liu et al., based on a piezo-electric polymer film sensor applied in a form of a mattress, showed a 97% accuracy in detecting four major positions during sleep on a group of 11 healthy participants [38] Few radar systems have been described in the literature. No data regarding the feasibility of automatic sleep position detection were found in these papers [39,40,41,42].

Contact sensors are ones attached to a patient’s body, and the placement of the sensor on the body is crucial to what signals could be detected. Contact sensors can be wrist or finger, chest, neck, or forehead devices. The most common contact sensors currently used in sleep evaluation are wrist/finger wearables. Most of these measure heart rate, heart rate variability, SaO2, and accelerometric signals. It has to be emphasized that this placement of the accelerometer does not allow accurate evaluation of the sleep position, as the trunk orientation does not depend on the forearm orientation. Current commercial wristband products, such as MI Band, Garmin Smartwatch, and Apple Watch, cannot recognize sleep positions [43]. A study by Yeng and colleagues showed that correct classification of sleep position was achieved in up to 85% of recording time with a wrist sensor, but the study was performed only on two participants. When placing an accelerometer on the chest, the accuracy in detecting sleep positions is very high. This was also shown in studies with the use of smartphone accelerometry. Two such studies showed accuracy in detecting sleep position as high as 97% and 95.9% on 6 and 19 subjects, respectively, [22,23].

Our previous study showed a 97.3% accuracy in distinguishing between supine and non-supine positions on 30 patients. Here, we confirmed about 97% in supine and non-supine position accuracies and at least 97.3% in other body position detection. We suggest the suprasternal notch is a perfect candidate to mount a small wireless sensor for sleep study purposes, as this place enables perfect detection of the acoustic signal related to breathing and a heart rhythm. Furthermore, as was shown in this study, sleep position can be accurately detected from this location. Moreover, this location of the sensor should not discourage patients from adopting a prone sleep position as this might be a problem with mounting the sensor on the chest.

## 5. Conclusions

Our study showed that placing the position sensor on the neck, at the suprasternal notch, may be highly effective in detecting sleep positions. We showed an accuracy above 97% for detecting each of four gross positions in a large cohort of patients. As this placement of the sensor might be less inconvenient than the chest placement, we feel the suprasternal notch is a good candidate for a single sensor placement in sleep studies.

## Figures and Tables

**Table 1:** Baseline characteristics of study participants and polysomnography data.

Characteristic	Males	Females	Total Patients
*N*	68	21	89
Age (years)	50.15±11.78	53.62±11.93	50.97±11.91
BMI (kg/m^2^)	30.09±4.91	29.35±6.04	29.91±5.21
TST (min)	386.21±49.09	399.66±50.54	389.38±49.76
Sleep efficiency * (%)	91.58±41.49	87.45±6.76	90.60±36.45
AHI (events/h)	37.12±24.12	28.45±17.06	35.08±22.95
AHI supine (events/h)	50.03±25.47	45.42±28.72	48.94±26.34
AHI non-supine (events/h)	25.78±26.17	14.82±14.77	23.19±24.42
Supine position in TST (min)	166.31±107.02	194.84±100.21	173.04±106.15
Supine position in TST (%)	43.70±27.79	48.82±24.26	44.91±27.08
Non-supine position in TST (min)	212.08±109.67	199.40±99.01	209.09±107.38
Non-supine position in TST (%)	55.35±27.52	50.29±24.78	54.16±26.98

Values were presented as mean ± standard deviation. BMI = body mass index, TST = total sleep time, TRT = total recording time, AHI = apnea–hypopnea index; * Calculated as (TST/TRT × 100%).

**Table 2:** Characterictics of OSA patients.

Characteristic	Males	Females	Total Patients
OSA patients *; *n* (% from specific group)	67 (98.5)	18 (85.7)	85 (95.5)
Supine OSA patients **; *n* (%)	34 (38.2)	13 (14.6)	47 (52.8)
Supine-isolated OSA patients ***; *n* (%)	5 (5.6)	6 (6.7)	11 (12.4)
Supine-predominant OSA patients ****; *n* (%)	29 (32.6)	7 (7.9)	36 (40.5)

* Patients with AHI ≥ 5; ** OSA patients with supine to non-supine sleep ratio of more than 2; *** positional OSA patients with non-supine AHI < 5, who slept at least 15 min both supine and non-supine; **** positional OSA patients with non-supine AHI ≥ 5; OSA—Obstructive sleep apnea.

## Data Availability

Data supporting the reported results are available from the corresponding author on reasonable request.
